# OA16 A great imitator of scleroderma

**DOI:** 10.1093/rap/rkac066.016

**Published:** 2022-09-28

**Authors:** Alexa Josè, Escudero Siosi, Thomas Gibbs, Shaheen Sultana, Joel David

**Affiliations:** Oxford University Hospitals NHS Foundation Trust, Oxford, United Kingdom; Oxford University Hospitals NHS Foundation Trust, Oxford, United Kingdom; Oxford University Hospitals NHS Foundation Trust, Oxford, United Kingdom; Oxford University Hospitals NHS Foundation Trust, Oxford, United Kingdom; Oxford University Hospitals NHS Foundation Trust, Oxford, United Kingdom

## Abstract

**Introduction/Background:**

Eosinophilic fasciitis is a rare disease of idiopathic aetiology, which can often mimic scleroderma in presentation and has been associated with haematological diseases. We present a case of eosinophilic fasciitis where the diagnosis was delayed due to the attribution of skin manifestations to scleroderma. This case highlights the importance of careful history and examination to recognize these differential diagnoses, prevent delay and improve outcome.

**Description/Method:**

A 69-year-old gentleman was referred to the Rheumatology clinic with suspected diffuse cutaneous systemic sclerosis. He presented with a 9-month history of skin tightening, pain and swelling affecting his legs, finding it difficult to fully extend at the knees, to drive or walk. His symptoms progressed overtime with skin changes spreading to all four extremities, abdomen and back. He reported weight loss of 12Kg associated with early satiety. He also experienced intermittent numbness of his hands. There were no respiratory symptoms, dysphagia or Raynaud’s phenomenon reported. His medical history includes atrial fibrillation and asbestos exposure. Currently on Apixaban 5mg/daily and Bisoprolol 2.5mg/daily.

Physical examination revealed diffuse brown hyperpigmentation, erythema and thickened skin on his upper and lower limbs, abdomen and back, it was difficult to crease the skin and there was ‘peau d’orange’ appearance in his forearms. His face and hands were spared. Phalen's and Tinel’s tests were negative.

Prior to clinical review he was investigated for paraneoplastic disease: a CT chest, abdomen and pelvis showed an incidental cyst in the thymus and emphysema with no underlying malignancy. Laboratory tests showed normocytic normochromic anaemia with haemoglobin 112 g/L and eosinophilia 1.5 x 10^9^/L, elevated erythrocyte sedimentation rate 62mm/h, CRP 57mg/L, polyclonal hypergammaglobulinaemia IgA 4.3g/L and IgG 24.85g/L. The myeloma screen were negative.

The absence of Raynaud’s, and the distribution of skin changes sparing his face, hands and feet, eosinophilia and raised inflammatory markers, made the diagnosis of eosinophilic fasciitis. Histological analysis of a full thickness skin biopsy confirmed patchy lymphoplasmacytic inflammatory cell infiltrate within the fascia and spilling into subcutis, with eosinophils.

Subsequently, he was commenced on prednisolone 40mg/daily reducing regimen and Methotrexate 20mg/weekly, with a good response and regression of the swelling and pain. He is now able to walk without difficulty.

**Discussion/Results:**

Eosinophilic fasciitis is rare. Symptoms can be similar to those of scleroderma, sometimes considered a variant. The exact aetiology is unknown; however, the literature mentions a potential paraneoplastic, inflammatory, infectious or physical trigger, leading to an abnormal response with accumulation of eosinophils in different tissues. Consequently, this may release transforming growth factor beta to activate fibroblasts, increasing expression of other factors in the connective tissue, resulting in fibrosis.

This case highlights that eosinophilic fasciitis present with hardening and thickening of the skin and surrounding tissue similar to that of scleroderma, however, lacks Raynaud’s phenomenon, sclerodactyly. Another striking features from his examination which helped us distinguishing from scleroderma was that his skin changes spare his face, hands and feet.

Carpal tunnel syndrome is described in the literature which can progress if untreated.

Typical blood tests findings documented in the literature include eosinophilia in 80% of the cases, elevated ESR and C-reactive protein (CRP), polyclonal hypergammaglobulinaemia, are usually present, however, eosinophilia may be transient, therefore normal eosinophil count does not exclude the disease. ANA and Rheumatoid factor can be positive in up to 10 % of the cases.

MRI of the extremities to look for thickening of the fascia has been used in other cases, however, it was not required in our case because our Orthopaedics colleagues helped us with performing an urgent full thickness biopsy.

Recommended treatment includes high dose steroids and immunosuppressive medication such Methotrexate is most often used for severe or resistant cases.

The prognosis is good, however if treatment is delayed patients can develop joint contractures in 85% of patients and skin hardness may persist.

In conclusion, careful history and examination is essential in differentiating scleroderma from the scleroderma-mimics especially when there is an atypical distribution, no sclerodactyly and no autoantibodies.

**Key learning points/Conclusion:**

1. This case suggest that clinicians should be aware that eosinophilic fasciitis typically spare the hands, feet and face.

2. Eosinophilia may be transient, therefore normal count does not exclude the disease.

3. Recognizing this rare but great imitator of scleroderma set the stage for prompt therapy in either condition and reduce morbidity.

